# Low prevalence of human papillomavirus in head and neck squamous cell carcinoma in the northwest region of the Philippines

**DOI:** 10.1371/journal.pone.0172240

**Published:** 2017-02-15

**Authors:** Pia Marie Albano, Dana Holzinger, Christianne Salvador, Jose Orosa, Sheryl Racelis, Modesty Leaño, Danilo Sanchez, Lara Mae Angeles, Gordana Halec, Markus Schmitt, John Donnie Ramos, Michael Pawlita

**Affiliations:** 1 Division of Molecular Diagnostics of Oncogenic Infections, Research Program Infection and Cancer, German Cancer Research Center (DKFZ), Heidelberg, Germany; 2 Department of Biology, College of Science, University of Santo Tomas, Manila, Philippines; 3 Research Center for the Natural and Applied Sciences, University of Santo Tomas, Manila, Philippines; 4 Deparment of ENT Head and Neck Surgery, Mariano Marcos Memorial Hospital and Medical Center, Ilocos Norte, Philippines; 5 Department of Pathology and Laboratories, Mariano Marcos Memorial Hospital and Medical Center, Ilocos Norte, Philippines; 6 Department of Clinical Pathology, University of Santo Tomas Hospital, Manila, Philippines; Universidad de Chile, CHILE

## Abstract

**Background:**

Geographic heterogeneity of human papillomavirus (HPV) involvement in head and neck squamous cell carcinoma (HNSCC) has been observed over the last few years. This trend has not been evaluated in the Philippines. Hence, this study aims to provide for the first time a data on the prevalence of HPV in HNSCC in the northwestern region of the Philippines.

**Methods:**

Two hundred one (201) biopsy samples (179 formalin fixed paraffin embedded and 22 fresh frozen) from 163 Filipino HNSCC cases (oral cavity = 88; larynx = 60; oropharynx = 15) diagnosed between 2003 to 2013 were initially included in this study. HPV DNA was detected by two methods: (1) BSGP5+/6+-PCR/ multiplex human papillomavirus genotyping and (2) TaqMan probes-based real-time qPCR. Presence of HPV type-specific transcripts were also analyzed by reverse transcription-PCR with subsequent hybridization to oligonucleotide probes coupled to Luminex beads. Co-amplification of the β-globin and ubiquitin C genes served as internal positive controls for DNA and RNA analyses, respectively.

**Results and conclusions:**

Of the 163, 82 (50.3%) cases had at least one tissue sample that was valid for molecular analysis. Only two of the DNA valid cases (2.4%) were HPV DNA-positive (HPV11 and HPV33). All HPV mRNA assays rendered negative results except for HPV11 transcripts. Results of this study may indicate that there is probably very low prevalence of HPV-associated HNSCC among Filipino adults living in a rural region of the Philippines. This study could serve as a benchmark for designing follow-up studies that would assess possible changes in trends of HNSCC among Filipinos in different ethnic regions of the country, especially urban areas in which the population is expected to adapt Western style sexual behavior. A prospective sampling of fresh frozen tissue is also highly recommended to ensure better molecular analyses.

## Introduction

Head and neck cancers (HNC) are neoplasms that arise in the oral cavity, nasopharynx, oropharynx, hypopharynx, larynx, paranasal sinuses, and salivary glands [1]. They are among the world’s most common cancers, with ∼599,637 new cases and ∼324,794 deaths from HNC (excluding nasopharyngeal cancer) occurring yearly [2]. Large scale epidemiological studies have shown HNC to be more common among men than among women. Moreover, about 90% of HNC cases are squamous cell carcinoma in origin, rendering it the most common histological type [1].

In the Philippines, annual incidence rates for laryngeal, oral cavity and oropharyngeal cancers are at 1.5, 2.4, and 1.3 per 100,000 population, respectively. Cancers of the lip/oral cavity, larynx, and pharynx rank 7^th^, 10^th^, and 13^th^, respectively, as most common malignancies among men while lip/oral cavity cancer as 10^th^ in incidence among women [2]. Tobacco and heavy alcohol use have been established as main risk factors in head and neck squamous cell carcinoma (HNSCC). Over the recent decades, effective campaigns against tobacco and alcohol use in many western countries have resulted in significant decline in the incidence of laryngeal and oral cavity but not oropharyngeal cancer [3, 4]. In contrast, a steady increase in the incidence of oropharyngeal squamous cell carcinoma (OPSCC) has been observed especially in the more economically developed countries, with human papillomavirus (HPV) infection as the preeminent risk factor [3, 5].

A systematic review and meta-analysis on the global prevalence and type distribution of HPV in HNSCC showed that HPV DNA was more prevalent in the oropharynx (45.8%) than larynx/hypopharynx (22.1%) and oral cavity (24.2%). HPV16 accounted for 82.2% of all HPV DNA-positive cases, making it the most commonly found genotype in HNSCC [6].

The relative prevalence of HPV-associated OPSCC also varies substantially among geographical regions, being highest (29%-93%) among cases from economically developed countries [3, 7]. As HPV, the primary and essential etiologic agent of cervical cancer, has been shown to be sexually transmitted, HPV-positive OPSCC has also been associated with sexual behavior [8–11].

Given the wide geographic variation of HPV involvement in HNSCC, this study evaluated for the first time the prevalence of HPV in HNSCC cases from the northwestern region of the Philippines diagnosed between 2003 to 2013.

## Materials and methods

### Clinical specimens and nucleic acid extraction

Ethical clearance was obtained from the study base, Mariano Marcos Memorial Hospital and Medical Center (MMMH-MC) in Ilocos Norte, Philippines. All participants gave their written informed consent. Formalin fixed paraffin embedded (FFPE) or fresh frozen biopsies from patients with histologically confirmed primary tumors of the head and neck seen at MMMH-MC between January 2003 to September 2013 were utilized in this study. Tissue sectioning, assessment of tumor content, and molecular analyses were performed at the German Cancer Research Center in Heidelberg, Germany.

FFPE and fresh frozen tissue sectioning was performed as described [12, 13]. Genomic DNA from the fresh frozen sections was extracted by MagNA Pure 96 DNA and viral NA Large Volume Kit (Roche, Penzberg, Germany) following the manufacturer’s recommendations. DNA extraction from FFPE sections was done as described [13, 14]. Total RNA was isolated from the fresh frozen and FFPE sections using the RNeasy Minikit (Qiagen, Hilden, Germany) and Pure Link FFPE Total RNA Isolation Kit (Invitrogen, Carlsbad, CA), respectively. DNAse I digestion was performed prior to the last washing step to ensure exclusive amplification of RNA [14].

### HPV DNA detection

All DNA extracts were analyzed for HPV DNA by broad spectrum general primer 5+/6+-polymerase chain reaction/multiplex human papillomavirus genotyping (BSGP5+/6+-PCR/MPG). Hybridization to HPV type-specific oligonucleotide probes coupled to fluorescence-labeled polystyrene beads (Luminex^®^ xMAP suspension array technology) was used to detect the labelled PCR products [15–17]. A 208-bp sequence of the β-globin gene served as internal positive control. Results were expressed as mean fluorescence intensity (MFI) of at least 50 beads per set and net MFI values >5 were considered positive [15, 16].

All tissue samples were also subjected to a second method of HPV16 and 18 DNA detection, a TaqMan probes-based real-time quantitative PCR (qPCR) targeting E6 gene sequences. β-globin was co-amplified to determine DNA quality and quantity. The predefined cut-off for high viral load was 0.5 HPV genome copies/cell, and samples with less than 20 cells (cut off: 40 beta-globin copies) were considered as invalid samples. The analytical sensitivity of this qPCR is less than 100 HPV16 or 18 genome copies [18].

### HPV mRNA analysis

The HPV DNA positives and a group of HPV DNA negatives (all OPSCC and randomly selected non-OPSCC) were analyzed for HPV16 E6*I mRNA and for other HPV types positive by HPV genotyping. Reverse transcription-PCR (RT-PCR) was performed using QuantiTect Virus Kit (Qiagen, Hilden, Germany) and type- and splice site-specific primers to amplify cDNA [12, 13]. The 65 to 75-bp biotinylated cDNA-PCR products were subsequently hybridized to oligonucleotide probes coupled to fluorescence-labeled polystyrene beads (Luminex^®^ suspension array technology) detectable with Luminex^®^ readers. Results were expressed as MFI of at least 50 beads per set and net MFI values >5 were considered positive. For HPV11, which lacks the E6*I splice site, a 77-bp cDNA sequence of its E6 full-length (fl) RNA was amplified and detected by hybridization to an internal oligonucleotide probe. Cellular ubiquitin C gene (ubC) was used to evaluate the quality of the recovered total RNA [13, 19].

### Statistical analysis

Since many of the samples had poor quality DNA, chi square test of homogeneity was performed using SPSS version 21 with a level of significance of 0.05 to verify whether the number of DNA valid cases were representative of the total number of cases initially analyzed.

## Results

A total of 221 biopsy samples (199 FFPE from 152 patients and 22 fresh frozen from 22 patients) were initially analyzed for presence of identifiable tumor cells. Hematoxylin and eosin (H&E) staining confirmed presence of tumor cells in all (n = 22) fresh frozen biopsies. Of the 199 FFPE tissue blocks, 179 samples from 141 patients still contained tumor cells.

All 22 fresh frozen tissues showed cellular β-globin co-amplification, signifying good DNA quality of the samples. Only 60 (42.6%) among the 141 patients had at least one FFPE block that was positive for β-globin. Nevertheless, chi-square test of homogeneity generated non-significant p-values for all clinical and epidemiological risk variables, indicating similar distribution between the groups—cases with valid DNA (n = 82) and cases with invalid DNA (n = 81, Table 1). Among the 82 DNA valid cases, only 13.4% were OPSCC cases and oral cavity squamous cell carcinoma (OSCC) was the most common at 53.7%. Median age at diagnosis was 60 years.

**Table 1:** Clinical and epidemiologic risk profile of the cases.

Characteristic	Category	All cases		Cases with valid DNA		Cases with invalid DNA		p value*
		n = 163	*(%)*	n = 82	*(%)*	n = 81	*(%)*	
Gender								0.085
	Male	110	*67*.*5*	64	*78*.*0*	46	*56*.*8*	
	Female	53	*32*.*5*	18	*22*.*0*	35	*43*.*2*	
Age at initial diagnosis (year)								0.624
	Median	61.5		60.0		63.0		
	*(Range)*	*(40–94)*		*(41–87)*		*(40–94)*		
	≤50	26	*16*.*0*	14	*17*.*1*	12	*14*.*8*	
	51–70	94	*57*.*7*	51	*62*.*2*	43	*53*.*1*	
	≥71	43	*26*.*4*	17	*20*.*7*	26	*32*.*1*	
Tumor site								0.761
	Oral cavity	88	*54*.*0*	44	*53*.*7*	44	*54*.*3*	
	Oropharynx	15	*9*.*2*	11	*13*.*4*	4	*4*.*9*	
	Larynx	60	*36*.*8*	27	*32*.*9*	33	*40*.*7*	
Tumor stage								0.440
	Tis, T1, & T2	52	*32*.*3*	29	*35*.*8*	23	*28*.*8*	
	T3 & T4	109	*67*.*7*	52	*64*.*2*	57	*71*.*3*	
	No information	2		1		1		
Tumor grade								0.930
	Well differentiated	119	*73*.*0*	58	*70*.*7*	61	*75*.*3*	
	Moderately or poorly differentiated	44	*27*.*0*	24	*29*.*3*	20	*24*.*7*	
Alcohol consumption								0.576
	Ever	55	*41*.*4*	35	*47*.*3*	20	*33*.*9*	
	Never	78	*58*.*6*	39	*52*.*7*	39	*66*.*1*	
	No information	30		8		22		
Duration of drinking (years)								0.275
	Median	30		30		32.5		
	*Range*	*(1–50)*		*(1–50)*		*(5–50)*		
Tobacco use								0.296
	Ever	79	*59*.*4*	52	*70*.*3*	27	*45*.*8*	
	Never	54	*40*.*6*	22	*29*.*7*	32	*54*.*2*	
	No information	30		8		22		
Pack years								0.328
	Mean	13		17		9.3		
	*Range*	*(1–60)*		*(1–60)*		*(3–50)*		

*Chi-square test

None of the samples tested positive to HPV16 or HPV18 DNA. However, two (2.4%) of the cases, both of which were laryngeal squamous cell carcinoma (LSCC), tested positive for HPV DNA—one HPV11 and one HPV33. HPV11 DNA was detected in both FFPE and the corresponding fresh frozen tissue samples, which were both available for molecular analyses. HPV33 DNA was detected in FFPE biopsy specimen.

All RNA extracts (n = 26; 11 OPSCC cases and 15 non-OPSCC cases) tested positive for Ubiquitin C (ubC) transcripts, proving good quality of the samples. However, none of them tested positive for the HPV16 E6*I transcripts. Other HPV type-specific transcripts were seen in the HPV11 DNA positive sample only (Table 2).

**Table 2:** Results of molecular analyses.

	Tumor Site		
	Oral Cavity	Larynx	Oropharynx
Cases with identifiable tumor cells in their samples (n = 163)	88	60	15
β-globin positive (n = 82)	44	27	11
HPV DNA positive	0	1-HPV33; 1-HPV11	0
HPV16 E6*I mRNA positive	0	0	0
Other HPV type-specific mRNA positive	0	1-HPV11	0

## Discussion

The current study shows that prevalence of HPV DNA in HNSCC among Filipinos living in the rural area is very low (2.4%), contrary to what has been observed in the more economically developed countries [3, 7]. Results suggest that HNSCC due to HPV may not yet be a considerable health burden to Filipinos living outside the metropolitan cities.

In the United States, approximately 60–70% of their OPSCC cases are HPV-related [3]. Among the northern European territories, Sweden recorded the highest incidence of HPV-associated cancer of the tonsil (93%) and base of tongue (84%) [20]. Diverse prevalence rates of HPV DNA in OPSCC were observed in South Wales, United Kingdom (55%), Germany (50%), France (46.5%), Australia (36%), and Netherlands (29%) [14, 21–24]. In Japan and North India, HPV DNA was present in 29.3% and 22.8% of their HNSCC cases, respectively [25, 26].

In contrast, findings of this study are similar to North-East Italy (HNSCC-6%; OPSCC-20%), Latin America and Central Europe (HNSCC-3.1%), Northern Spain (OPSCC-3.2%), and Senegal and Nigeria of Africa (HNSCC-0 to 3.4%) [18, 27–30].

It can be observed that prevalence of OPSCC in the northwest region of the Philippines was noticeably low, accounting for only about 13.4% of the HNSCC cases. It has been shown that in countries or regions where there was increasing prevalence of HPV-associated HNSCC, there was likewise parallel increasing incidence of OPSCC[3]. In the Philippines, no significant increase in the incidence of OPSCC nor significant differences in the incidence trends between OPSCC and OSCC have been noted in the recent years [3].

HPV-associated HNSCC has also been observed to be more apparent among younger cohorts (<45 years old), usually without history of alcohol or tobacco use [31–34]. In contrast, majority (82.9%) of the cases here were above 51 years of age; 70.3% were ever tobacco smokers; 47.3% have used alcohol during their lifetime; and 42.4% have used both tobacco and alcohol. These observations suggest that tobacco and alcohol may still be the major etiologic agents of HNSCC in northwest Philippines and not HPV [7, 35, 36].

The Philippines has been identified as among the nations with high tobacco use but weak control programs [37]. Coincidentally, northwest Philippines happens to be the biggest tobacco producing region of the country. Some cases even engaged in betel nut chewing, tobacco chewing, and/or reverse smoking, which are habits proven to be risk factors for oral cavity cancer [38, 39].

There is now sufficient evidence for a causal role of HPV in HNSCC, in which risk factors include history of ever having oral sex, greater numbers of sexual partners, and a history of same-sex contact [40–42]. Oral sex is considered "dirty and unnatural" by most Filipinos especially the older and lower-class living in the rural areas. It was even suggested that only 10–15% of this population practiced oral sex, and they are mostly the modern Filipino youth living in metropolitan area where sexual practices are more evolved [43]. The characteristics of the cases in this study—older, living in the rural areas, and conservative in terms of sexual behavior—may support the very low prevalence of HPV-driven OPSCC in the region.

To ensure reproducible results, rigorous molecular analyses were done, with the inclusion of the appropriate controls and cleaning protocols to prevent cross-contamination. For instance, a second independent assay targeting a different HPV sequence and shorter amplicons than the BSGP5+/6+ PCR was performed. This TaqMan probes-based real-time qPCR was for the simultaneous detection of shorter amplicons of the HPV16 and 18 E6 genes (∼104bp), and the β-globin housekeeping gene (∼110bp) [18]. This study focused on HPV16 and 18 to exclude that these frequently detected transforming types have been missed due to degradation or chemical modification because of formalin fixation and paraffin embedding. The qPCR assay did not detect HPV 16 or 18 DNA in any of the FFPE or fresh frozen samples. No similar assay for the other HPV types were available.

Interestingly, one of the LSCC cases, a 49-year old male with history of recurrent respiratory papillomatosis (RRP) tested positive for both HPV11 DNA and E6 mRNA. RRP, a disease characterized by presence of squamous wart-like lesions within the respiratory tract, is caused by the ‘‘low risk” HPV types 6 and 11 [44]. Rare cases of progression from RRP to invasive LSCC have been reported earlier [45–47]. The integration of HPV6 and 11 DNA into host genome, leading to the production of virus-cellular fusion transcripts have been demonstrated in said studies [45–48]. In view of the overall poor DNA quality of our samples, we did not attempt to analyze viral integration.

HPV16 E6*I mRNA was further analyzed in all OPSCC and random non-OPSCC cases. The samples showed high ubC RNA validity due to the extremely short amplicons applied. All OPSCC and only 15 randomly selected non-OPSCC cases were included in the analysis given that pooled HPV DNA prevalence estimates was highest for oropharynx at 45.8% and only 22.1% and 24.2% for larynx (including hypopharynx) and oral cavity, respectively [6]. HPV16 E6*I mRNA, among other HPV types, was analyzed in all OPSCC and random non-OPSCC cases since it has been shown to account for 82.2% of all HPV DNA positive cases [6]. The very low prevalence of HPV DNA is corroborated by the absence of HPV16 E6*I mRNA in the samples.

The HPV mRNA reverse transcription–PCR assay described here is more sensitive than the BSGP5+/6+-PCR/MPG or real-time qPCR as it can detect ≤100 copies of 65-75bp of mRNA per reaction [12]. One of the cases tested positive for HPV33 DNA but negative for HPV33 E6*1 mRNA. A study on the prevalence of HPV in invasive laryngeal cancer in the United States showed that HPV16 and HPV33 were the most commonly detected types. The said study, however, did not analyze for HPV33 mRNA [49]. HPV transcripts are more specific markers of transformation and their detection is still considered the gold standard in assessing HPV genome expression and viral activity in the infected cells [50]. Halec et al. likewise reported presence of HPV33 DNA in two of their 32 LSCC samples but detected no HPV33 E6*1 mRNA [13]. Due to the absence of HPV33 E6*1 mRNA in the HPV33 DNA positive case, it can be hypothesized that the HPV33 DNA positivity could have resulted from infection in tumor or adjacent tissue, or even HPV debris in oral HPV infections [13].

This study has several limitations. Similar to one conducted on samples from Nigeria [27], many of the available FFPE blocks were not suitable for molecular analyses. For lack of facilities and financial constraints, it is not the practice among hospitals and clinics in the Philippines to collect and store fresh frozen tissues for prospective molecular analyses. Hence, only FFPE specimens were available for analysis until this study was initiated. This single-institution study, which focused on a rural region of the Philippines, cannot represent the entirety of Filipino HNSCC cases since epidemiologic risk factors may differ entirely between rural and urban dwellers.

Therefore, the results of this study should be interpreted cautiously. Molecular analyses were quite challenging due to the poor quality of the specimens. Attempts to improve the quality of the FFPE-derived DNA were done but not successful. Therefore, a follow-up study is recommended to assess possible changes in trends of HNSCC among Filipinos in different ethnic regions of the country, especially urban areas in which the population is expected to adapt Western style sexual behavior. A well-structured prospective case control study involving a greater number of OPSCC cases from both rural and urbanized areas, preferably using fresh frozen tissue for molecular analyses is being proposed.
